# Efficacy and Safety of the Natural Killer T Cell–Stimulatory Glycolipid OCH-NCNP1 for Patients With Relapsing Multiple Sclerosis: Protocol for a Randomized Placebo-Controlled Clinical Trial

**DOI:** 10.2196/46709

**Published:** 2024-01-15

**Authors:** Tomoko Okamoto, Takami Ishizuka, Reiko Shimizu, Yasuko Asahina, Harumasa Nakamura, Yuko Shimizu, Yoichiro Nishida, Takanori Yokota, Youwei Lin, Wakiro Sato, Takashi Yamamura

**Affiliations:** 1 Department of Neurology National Center Hospital National Center of Neurology and Psychiatry Tokyo Japan; 2 Department of Clinical Research Support Clinical Research and Education Promotion Division, National Center Hospital National Center of Neurology and Psychiatry Tokyo Japan; 3 Department of Neurology Tokyo Women's Medical University Tokyo Japan; 4 Department of Neurology and Neurological Science Graduate School of Medical and Dental Sciences Tokyo Medical and Dental University Tokyo Japan; 5 Department of Immunology National Institute of Neuroscience National Center of Neurology and Psychiatry Tokyo Japan

**Keywords:** OCH-NCNP1, natural killer cell, multiple sclerosis, clinical study, randomized controlled trial, autoimmune inflammatory disease, degeneration, clinical efficacy, biomarker, relapse, disability, imaging, autoimmune, RCT, Expanded Disability Status Scale, EDSS, neuromyelitis optica, optic neuritis, acute myelitis, Fisher exact test, MRI, magnetic resonance imaging, myelin sheath, demyelinating lesion, aminotransferase, clinicopathology, pathology

## Abstract

**Background:**

Multiple sclerosis (MS) is an autoimmune inflammatory disease of the central nervous system that causes myelin sheath damage and axonal degeneration. The glycolipid (2S, 3*S*, 4*R*)-1-O-(α-d-galactosyl)-2-tetracosanoylamino-1,3,4-nonaetriol (OCH-NCNP1 or OCH) exerts an immunoregulatory action that suppresses T helper (Th)1 cell–mediated immune responses through natural killer T cell activation, selective interleukin-4 production, and Th2 bias induction in human CD4-positive natural killer T cells.

**Objective:**

This trial aims to investigate the efficacy and safety of the immunomodulator OCH in patients with relapsing MS through 24-week repeated administration.

**Methods:**

This protocol describes a double-blind, multicenter, placebo-controlled, randomized phase II clinical trial that was initiated in September 2019. The participants were randomly assigned to either a placebo control group or an OCH-NCNP1 group and the investigational drug (3.0 mg) was orally administered once weekly for the 24-week duration. Major inclusion criteria are as follows: patients had been diagnosed with relapsing MS (relapsing-remitting and/or secondary progressive MS) based on the revised McDonald criteria or were diagnosed with MS by an attending physician as noted in their medical records; patients with at least two medically confirmed clinical exacerbations within 24 months prior to consent or one exacerbation within 12 months prior to consent; patients with at least one lesion suspected to be MS on screening magnetic resonance imaging (MRI); and patients with 7 points or less in the Expanded Disability Status Scale during screening. Major exclusion criteria are as follows: diagnosis of neuromyelitis optica and one of optic neuritis, acute myelitis, and satisfying at least two of the following three items: (1) spinal cord MRI lesion extending across at least three vertebral bodies, (2) no brain MRI lesions during onset (at least four cerebral white matter lesions or three lesions, one of which is around the lateral ventricle), and (3) neuromyelitis optica–immunoglobulin G or antiaquaporin-4 antibody-positive. Outcome measures include the primary outcome of MRI changes (the percentage of subjects with new or newly expanded lesions at 24 weeks on T2-weighted MRI) and the secondary outcomes annual relapse rate (number of recurrences per year), relapse-free period (time to recurrence), sustained reduction in disability (SRD) occurrence rate, period until SRD (time to SRD occurrence), no evidence of disease activity, and exploratory biomarkers from phase I trials (such as gene expression, cell frequency, and intestinal and oral microbiome).

**Results:**

We plan to enroll 30 patients in the full analysis set. Enrollment was closed in June 2021 and the study analysis was completed in March 2023.

**Conclusions:**

This randomized controlled trial will determine whether OCH-NCNP1 is effective and safe in patients with MS as well as provide evidence for the potential of OCH-NCNP1 as a therapeutic agent for MS.

**Trial Registration:**

ClinicalTrials.gov NCT04211740; https://clinicaltrials.gov/study/NCT04211740

**International Registered Report Identifier (IRRID):**

DERR1-10.2196/46709

## Introduction

Multiple sclerosis (MS) is considered to be an autoimmune disease triggered by environmental factors that act on a genetically susceptible host. Both major histocompatibility complex (MHC) and non-MHC genes are risk factors for the development of MS. In addition, environmental factors such as low vitamin D, low ultraviolet radiation exposure, cigarette smoking, obesity, and Epstein-Barr virus exposure can increase the risk for both the development of MS and a more severe disease course. Accumulating evidence suggests that dysregulation of the intestinal microbiome (dysbiosis) constitutes an important factor contributing to MS pathogenesis. The microbiome regulates T cell function, with both regulatory and pathogenic effects, thereby playing an important role in autoimmune responses [1].

MS is a cell-mediated autoimmune disease directed against central nervous system (CNS) myelin antigens involving both CD4+ and CD8+ T cells, especially the so-called pathogenic T helper (Th)17- and Th1-type and CD8 myelin autoreactive T cells. The development of MS is likely triggered or promoted by breakdown of the delicate balance between autoreactive T cells and regulatory lymphocytes [1].

Although MS has historically been considered a demyelinating disease of the CNS and white matter, in recent years, neurodegeneration of the cortical and deep gray matter has been recognized to play a role in the pathogenesis of MS. Cortical atrophy is associated with disease progression, which has emerged as one of the best predictors of neurological disability in MS [1].

According to a recent worldwide epidemiological study, the number of patients with MS is estimated to be 2.8 million, which has been increasing in every world region since 2013 [2]. The estimated total economic burden of MS in the United States is US $85.4 billion, with a direct medical cost of US $63.3 billion and indirect and nonmedical costs of US $22.1 billion [3].

Relapsing-remitting MS (RRMS) initially involves clinical relapses with near or complete recovery; however, recovery over time may be incomplete and disability often worsens [4]. Approximately 20% of patients with RRMS develop a progressive form of MS accompanied by chronic neuroinflammation. Such cases are referred to as secondary progressive MS (SPMS). The term “relapsing MS” (RMS) is used to describe the condition of patients with repeated relapses of either RRMS or SPMS.

As understanding of the pathomechanism of MS progresses, various disease-modifying drugs have been used in clinical practice, including sphingosine-1-phosphate receptor modulators (fingolimod, siponimod), a monoclonal antibody that selectively binds the α4 integrin subunit (natalizumab), and CD20 monoclonal antibodies (ofatumumab, ocrelizumab), resulting in an overall improvement of patient prognosis. However, there are patients for whom current treatments are ineffective and there are cases where current treatments are intolerable due to side effects. Furthermore, progressive MS remains refractory to current drugs and constitutes an unmet medical need [4]. These observations highlight the need for new, safer therapeutic oral agents.

In 1997, Kawano et al [5] reported the discovery of sponge-derived α-galactosylceramide (α-GC) as a glycolipid ligand that stimulates natural killer T (NKT) cells. However, because this glycolipid stimulates NKT cells and induces the production of both interleukin (IL)-4 and interferon (IFN)-γ, a search for glycolipids that selectively induce IL-4 production was initiated to treat Th1 cytokine–dependent autoimmune diseases such as MS. These efforts led to the discovery of (2S, 3*S*, 4*R*)-1-O-(α-d-galactosyl)-2-tetracosanoylamino-1,3,4-nonaetriol (OCH-NCNP1; hereafter referred to as OCH), which was detected during screening for glycolipids that selectively induce the production of Th2 cytokines [6].

OCH is a derivative (synthetic glycolipid) of α-GC with a shortened fatty-acid chain (sphingosine chain). The compound is a white to slightly yellow powder that is extremely insoluble in methanol or ethanol and is practically insoluble in water. OCH exhibits an immunoregulatory action that suppresses Th1 cell–mediated immune responses through NKT cell activation, selective IL-4 production, and Th2 bias induction in human CD4+ NKT cells [7].

Although the mechanism by which OCH induces Th2 bias in NKT cells is not fully understood, it has been suggested to involve both cell intrinsic and extrinsic factors [8,9]. As a cell intrinsic mechanism, IFN-γ production by NKT cells was reported to be more susceptible to the sphingosine length of the glycolipid ligand compared to IL-4 production, and the length of the sphingosine chain determined the half-life of NKT cell stimulation by CD1d-associated glycolipids. As an extrinsic regulatory mechanism, OCH suppresses the production of IFN-γ, not only by NKT cells but also by NK cells, compared with that of α-GC. OCH induced lower IL-12 production due to ineffective primary IFN-γ and CD40 ligand expression by NKT cells, which resulted in lower secondary IFN-γ induction.

Animal studies verified that OCH can be administered orally to control Th1 cell–mediated autoimmune pathology in mice, with efficacy against autoimmune disease models such as experimental autoimmune encephalomyelitis, collagen-induced myelitis [10], nonobese diabetes [11], and inflammatory enteritis [12].

We here describe a protocol for a feasibility study that will be conducted in patients with RMS to investigate the efficacy and safety of OCH immunomodulators. Through 24 weeks of weekly administration, this trial was performed to confirm changes in exploratory T cell and NK cell biomarkers that fluctuated in the phase I trial [13], as well as measures of efficacy, including recurrence, dysfunction, and magnetic resonance imaging (MRI) changes, and their association with combined endpoints. In alignment with past clinical trials [14,15] and European Medical Agency guidelines, the primary outcome will be the percentage of subjects with new or newly expanded lesions at 24 weeks on T2-weighted MRIs.

## Methods

### Study Design

This is a double-blind, multicenter, placebo-controlled, randomized phase II clinical trial with a 24-week duration. This trial investigates the efficacy and safety of the immunomodulator OCH in patients with RMS through 24-week repeated administration. The protocol was also designed to confirm exploratory biomarkers of T cells and NK cells that fluctuated in the phase I trial [13], as well as efficacy-related clinical endpoints, including relapses, disability, and MRI changes, and their association with combined endpoints. Recruitment opened in September 2019 and concluded in June 2021, with participants randomly assigned to either a placebo control group or an OCH group using a clinical-based management system (Translational Research Center for Medical Innovation, Kobe, Japan). The researchers were blinded to the participant group assignments, and the packaging appearance of the control and investigational drugs was confirmed to be indistinguishable.

An independent data monitoring committee will regularly audit all available data, including safety, and recommend trial continuation to the principal investigator.

### Selection Criteria

Inclusion and exclusion criteria for this study are provided in Textbox 1.

**Inclusion and exclusion criteria.**

Inclusion criteria

- Patients who provided written consent for trial participation
- Patients had been diagnosed with relapsed multiple sclerosis (MS), including relapse-remitting MS and/or secondary progressive MS, based on the revised McDonald criteria or were diagnosed with MS by an attending physician as noted in their medical records
- Patients with at least two medically confirmed clinical exacerbations within 24 months prior to consent or one exacerbation within 12 months prior to consent
- Patients with at least one lesion suspected to be MS on screening magnetic resonance imaging (MRI)
- Patients with 7 points or less in the Expanded Disability Status Scale during screening
- Patients who were 20-65 years old at the time of consent
- Patients who can practice contraception until 90 days after final administration of the investigational drug
- Patients with no clinical or test findings suggesting acute recurrence based on evaluation by a neurologist

Exclusion criteria

- Diagnosis of neuromyelitis optica and one of the three following three criteria: optic neuritis, acute myelitis, and satisfied at least two of the following three items: (1) spinal cord MRI lesion extending across at least three vertebral bodies, (2) no brain MRI lesions during onset (at least four cerebral white matter lesions or three lesions, one of which is around the lateral ventricle), and (3) neuromyelitis optica–immunoglobulin G (IgG) or antiaquaporin-4 antibody-positive
- Currently pregnant or nursing
- Contraindication for MRI (eg, those with metal implants or a pacemaker) or cases in which MRI is difficult to perform (eg, claustrophobia)
- History of allergic or hypersensitive reaction to gadolinium contrast
- History of liver disease, including liver transplantation, viral hepatitis, autoimmune hepatitis, cirrhosis, and hepatic malignancies
- Liver dysfunction determined by the screening test or baseline test (alanine aminotransferase, aspartate aminotransferase, γ-guanosine triphosphate, or alkaline phosphatase) exceeding twice the upper limit of normal and total bilirubin exceeding 1.5-fold the upper limit of normal
- History of malignant tumors in the past 5 years (however, patients deemed to have no recurrence for at least 5 years prior to consent can be enrolled)
- Varicella-zoster virus IgG antibody–negative
- Positive for syphilis serum reaction (treponema pallidum latex agglutination, rapid plasma reagin)
- positive for β-D glucan (exceeding the standard value) or T-spot
- positive for antiaquaporin-4 antibody
- history of human immunodeficiency virus infection or who have been confirmed positive by a screening test
- History of hepatitis B infection (hepatitis B surface antigen–positive or hepatitis B core antibody–positive), or patients who have been confirmed positive by a screening test
- History of stem cell transplantation, organ transplantation, and treatment for rejection
- Physical, mental, or social condition affecting the ability to provide consent to or complete the trial
- Participation in other clinical trials that received the investigational drug within 4 months prior to enrollment
- Blood donation (200 mL within 2 months, 400 mL within 3 months) prior to enrollment
- Peripheral blood lymphocyte count <600/mm^3^ by screening or baseline examination
- With or suspected of having an infectious disease
- Immunocompromised patients
- Inflammatory bowel disease
- Use of the following prohibited concomitant therapies:
  interferon-β preparation (prohibited 1 month before trial enrollment); fingolimod hydrochloride (prohibited 6 months before trial enrollment); natalizumab (prohibited 3 months before trial enrollment); glatiramer acetate (prohibited 1 month before trial enrollment); dimethyl fumarate (prohibited 3 months before trial enrollment); drugs other than azathioprine that have immunosuppressive, immunostimulatory, or myelosuppressive effects (prohibited 3 months before trial enrollment); pulse therapy using corticosteroids (prohibited 1 month before clinical trial registration, except when MS recurs); plasmapheresis, immunoadsorption therapy, lymphocyte depletion therapy (prohibited from the time of consent acquisition); immunoglobulin preparation (prohibited 2 months before trial enrollment); vaccines (prohibited 1 month before trial enrollment, except when permitted by the investigator); other investigational drugs (prohibited 4 months before trial enrollment); nonsteroidal anti-inflammatory drug (liver damage at the time of screening should be checked, and for patients who take it regularly, this drug can be used in combination by fixing the dosage and administration. Additionally, when used for adverse events, it should be possible to use this drug within the minimum necessary range)

Additional exclusion criteria:

- Patients with evident prolongation of the QT/QTc interval prior to the trial (eg, patients with repeating QTc interval>450 ms)
- Patients with a history of other risk factors for torsades de pointes (eg, family history of heart failure, hypokalemia, and long QT syndrome)
- History of severe drug or food allergy
- Patients with drug or alcohol dependence in the past or present
- Asthma (excluding patients with no history of treatment or attack for at least 10 years)
- Diagnosis of epilepsy or patients with a history of seizures (excluding febrile seizures)
- Other pathological symptoms, illnesses, or history that may affect this trial
- Other factors resulting in unsuitability for this trial as deemed by the principal investigator; if such exclusion occurs, the specific rationale will be described in the trial results

### Sample Size Estimate

The target number of participants was 30 (15 per group) based on feasibility. The detection power was calculated when the primary outcome (proportion of subjects with new or enlarged existing lesions detected on T2-weighted images) was compared using the Fisher exact test. With 60% of patients placed in the placebo group based on the results of the domestic phase II study (D1201 study) at the time of fingolimod development [16] and assuming a 5% proportion in the OCH group, the detection power was 86.5%. Furthermore, when the OCH group was set to 10%, the detection power was 74.6% (the significance level was set to 5% using a two-tailed test).

### Patient Clinicodemographic Characteristics

The following clinicodemographic information was recorded for each participant: (1) subject background (eg, birth date, sex, body height, body weight); (2) urine pregnancy test; (3) infectious disease or antibody test; (4) vital signs (blood pressure, pulse rate, body temperature, and breathing rate); (5) neurological symptoms rating scale (Expanded Disability Status Scale [EDSS] or functional disability scale); (6) clinical tests (hematological test, hematobiochemical test, or urine test); (7) 12-lead electrocardiogram; (8) echocardiography; (9) chest or abdominal X-ray; (10) abdominal computed tomography; (11) MRI; (12) peripheral blood gene expression level (reverse transcription–polymerase chain reaction); (13) frequencies of NK, NKT, T cells, or other lymphocyte subsets; (14) frequencies of Th1, Th2, or Th17 cells; (15) intestinal and oral microbiome; (16) adverse events; (17) combination drugs and important nondrug therapies; and (18) Columbia Suicide Severity Rating Scale.

### Outcome Measurements and Study Timeline

The outcomes that will be measured repeatedly throughout the trial and the corresponding time points are shown in Multimedia Appendix 1. The main outcome is MRI changes (the percentage of subjects with new or newly expanded lesions on T2-weighted MRI). In addition, the annual relapse rate, cumulative number of new or newly expanded lesions on T2-weighted MRI, percentage of subjects who did not have lesions at 12 and 24 weeks on T2-weighted images of head MRI compared to those at the preobservation period at 24 weeks, brain atrophy in T1-3D images, percentage of subjects with no contrast-enhanced lesions on gadolinium-enhanced T1-weighted images, number of contrast-enhanced lesions on gadolinium-enhanced T1-weighted images, changes in volume of demyelinating lesions on fluid-attenuated inversion recovery 3D images, diffusion tensor imaging changes in myelin sheath lesions, and changes in myelin sheath lesions by a myelin map will be assessed. Additional outcomes include the relapse-free period (from randomization to earliest relapse), sustained reduction in disability occurrence rate, period until sustained reduction in disability (from randomization), no evidence of disease activity, and exploratory biomarkers from phase I trials (peripheral blood gene expression, frequencies of lymphocyte subsets, and intestinal and oral microbiome) [13].

### Adverse Events

Adverse events, defined as any undesired or unintended sign (including abnormal fluctuations in each test value), symptoms, or illness that occurs between the start of investigational drug administration and end or discontinuation of the investigational drug (regardless of its association with the study drug), will be recorded. The recordings will include the degree of symptoms on a 1-5 scale (1=asymptomatic or mild adverse events, 5=death due to the adverse event), outcomes from 1 to 6 (1=event disappeared or normalized, 6=death), and association with the investigational drug from 1 to 4 (1=associated, 4=not associated).

### Discontinuation Criteria

Discontinuation of involvement in the clinical trial may occur for any patient because of voluntary withdrawal or for any of the following reasons: (1) serious adverse events; (2) pregnancy; (3) alanine aminotransferase and aspartate aminotransferase >5-fold higher than the standard value upper limit; (4) total bilirubin exceeding 2.0 mg/dL; (5) number of peripheral blood lymphocytes <500/mm^3^ and administration of the investigational drug is stopped three times in a row without recovery, even after three tests; (6) occurrence of a grade-3 event based on Common Terminology Criteria for Adverse Events v4.0-JCOG; (7) new neurological symptoms with unpredictable MRI findings observed from the course of MS; (8) more than one recurrence of MS; (9) at least three courses of pulse steroid therapy performed; (10) an important management problem is discovered (subject noncompliance); (11) significant deviation from the protocol; (12) administration of the investigational drug is stopped at least three times, regardless of the reason; and (13) at the discretion of the principal investigator. If discontinuation occurs because of this final criterion, justification will be provided in the results report.

### Administration of Trial Compound

The investigational drug OCH (3.0 mg) in the form of granules (total of 0.3 g) or placebo granules alone (0.3 g) was orally administered with approximately 150 mL of water 30 min before breakfast once weekly for the 24-week trial duration. The white granules were composed of crystalline cellulose, mannitol, sodium croscarmellose, low-substituted hydroxypropyl cellulose, and polysorbate 80.

### Statistical Methods

Patient clinicopathological data will be summarized using descriptive statistics for each group. The proportion of subjects with new or enlarged existing lesions on T2-weighted MRIs in each group will be calculated and compared using the Fisher exact test with 95% CIs. The proportion of data between groups related to the primary endpoint will also be compared using the Fisher exact test with 95% CIs. For time-to-event data such as relapse-free periods, Kaplan-Meier estimates will be calculated for each group and groups will be compared using the log-rank test. The expression rates of safety-related outcomes and adverse events will be calculated for each group. For various test values, a list of measured values will be created for each group. The summary statistics for each group and measurement time point will also be calculated.

### Patient and Public Involvement

Patients were first involved in this study during the recruitment and screening processes. Based on previous experiences with patients with MS, the importance of the outcomes measured in this study was evaluated and determined based on their medical importance and impact on the patients’ quality of life. The patients recruited for the study agreed to the methods of disseminating the aggregate results when providing informed consent.

### Ethical Considerations

This study was conducted in compliance with the Declaration of Helsinki and all applicable local and national regulatory laws with approval from the National Center of Neurology and Psychiatry institutional review board (approval number II-013). The legal representatives of each patient provided written informed consent prior to participation in the study. Copayment reduction fees were paid to the trial participants in accordance with the regulations of the implementing medical institution.

### Dissemination

The results will be disseminated in peer-reviewed journals and presented at relevant conferences.

## Results

The first patient completed registration in December 2019 and the last patient completed registration in June 2021. The full analysis set comprised 30 cases and the study analysis was completed in March 2023. Preliminary analysis suggests that OCH may be effective for RMS (particularly SPMS).

## Discussion

### Principal Results

This will be the first randomized double-blind placebo-controlled trial to study the efficacy and safety of OCH. This randomized controlled trial will determine whether OCH is effective and safe in patients with MS. The results of this trial are expected to provide evidence for the potential of using OCH as a therapeutic agent for MS.

A single-dose trial (STEP 1) trial was conducted in healthy adults from November 2012 to July 2013 and a repeated-dose trial (STEP 2) was conducted in patients with RRMS from March 2014 to August 2017 [13]. In a phase I trial consisting of STEP 1 and STEP 2, OCH was administered to 28 patients (STEP 1: 3 patients×5 groups in all cohorts, STEP 2: 7 patients×1 group in a cohort, 3 patients×2 groups in a cohort).

Grade-1 adverse events and side effects were noted in STEP 1 and there were no serious adverse events or discontinuations. In STEP 2, serious side effects (acidosis and altered state of consciousness, depression, muscle weakness, and malaise) were reported in three patients in the 0.3 and 3 mg groups. Two patients discontinued treatment; however, all patients recovered. No other serious events were observed, confirming that the patients tolerated doses of 0.3, 1, and 3 mg. After OCH administration, there was no significant change in the neurological symptom evaluation scale score (EDSS or functional disability scale) even in STEP 2 because of the short observation period. MRI revealed clinically significant abnormalities in one patient in the 0.3 mg group; this patient discontinued the study.

A phase I physician-led clinical trial, conducted as an early exploratory clinical trial, determined that the dose was likely to have pharmacological action (fluctuations in some biomarkers) in humans. The dose at which pharmacological action was observed in experimental autoimmune encephalomyelitis model mice was 0.4-0.5 mg/kg. Considering that the area under the curve (AUC)_0-∞_ following oral administration of 5 mg/kg to mice was 2922 ng·h/mL, the AUC_0-∞_ following oral administration of 0.5 mg/kg (dose at which pharmacological action was observed) was estimated as 292.2 ng･h/mL. Assuming a correlation between systemic exposure (AUC_0-∞_) and pharmacological effects and systemic exposure in monkeys (AUC_0-∞_ after oral administration of 10 mg/kg was 2376, SD 1164 ng), pharmacological action may be expected in humans if at least approximately 1.2 mg/kg is orally administered.

In STEP 1 of the phase I trial, administration of OCH at doses of 0.3, 1, 3, 10, 30, 100, and 300 mg to healthy adults was planned. However, STEP 1 was completed at 30 mg; in the subsequent STEP 2, OCH at 0.3, 1, and 3 mg was administered to patients with MS. Importantly, no tolerability problems were noted.

The following changes in exploratory biomarkers were observed. In analysis of the immune cell subsets using flow cytometry, (1) inhibitory T cells (Foxp3+T cells) and effector regulatory T cells (CD45RA-FoxP3^high^ T cells) tended to increase and (2) granulocyte-macrophage colony-stimulating factor–producing Th cells transiently decreased in both healthy subjects and patients with MS. Recently, granulocyte-macrophage colony-stimulating factor–producing Th cells were identified as pathogenic cells in MS [17]. These changes suggest that oral OCH administration can correct the proinflammatory changes linked with disease activity in MS. Moreover, by conducting DNA microarray analysis of whole blood cells, we identified upregulation of several immunoregulatory genes and downregulation of several inflammatory genes in both healthy subjects and patients with MS, further supporting the immunoregulatory effect of OCH.

Based on the above safety profile and biomarker analysis results, a dose of 3 mg was selected as the investigational dose for this trial.

### Limitations

The primary limitation of our trial design is the small sample size. The sample size in this study was determined to be 30 participants based on the results of the previous phase I study. This study was limited to a small cohort of patients over a 24-week timeline and involved weekly administration of one dose of OCH. Although necessary in this early stage of the investigation, the small sample size could limit the ability to identify potential adverse events that may be rare or related to specific clinicodemographic traits of patients not captured in this study. However, the robust and clinically relevant nature of our primary outcome measure and sample size determined in prior studies are expected to provide indications of drug efficacy.

### Conclusions

This article describes an NKT cell–stimulatory glycolipid (OCH) protocol. This randomized controlled trial will determine whether OCH is effective and safe in patients with MS. The results of the trial are expected to provide evidence for the potential of OCH as a therapeutic agent for MS.

## Appendix

Multimedia Appendix 1 Observation, examination, and survey schedule.

## Abbreviations

α-GC: α-galactosylceramide
AUC: area under the curve
CNS: central nervous system
EDSS: expanded disability status scale
IFN: interferon
IL: interleukin
MHC: major histocompatibility complex
MRI: magnetic resonance imaging
MS: multiple sclerosis
NKT: natural killer T cells
OCH: (2S, 3S, 4R)-1-O-(α-d-galactosyl)-2-tetracosanoylamino-1,3,4-nonaetriol
RMS: relapsing multiple sclerosis
RRMS: relapsing-remitting multiple sclerosis
SPMS: secondary progressive multiple sclerosis
Th: T helper

## Data Availability

Data are not included in this protocol, but will be presented in an upcoming paper summarizing the results of this clinical trial. The data sets are available from the corresponding author upon reasonable request taking into account our patent strategy.
