# Supplementary material for: Efficacy and Safety of the Natural Killer T Cell–Stimulatory Glycolipid OCH-NCNP1 for Patients With Relapsing Multiple Sclerosis: Protocol for a Randomized Placebo-Controlled Clinical Trial
Source: JMIR Res Protoc. 2024 Jan 15;13:e46709. doi: 10.2196/46709 (PMC10825757; doi:10.2196/46709)
Supplement: Multimedia Appendix 1 [file resprot_v13i1e46709_app1.docx]

**Multimedia Appendix 1.** Observation, examination, and survey schedule.

| **Item** | **Screening period** | **Before administration** | **Administration period** | | | | | | | | **Postobservation (T9)** | **Discontinuation of administration** |
| --- | --- | --- | --- | --- | --- | --- | --- | --- | --- | --- | --- | --- |
|  | S1^a^ | B1 | T1 | T2 | T3 | T4 | T5 | T6 | T7 | T8 |  |  |
| **Number of days from the start date of administration**  **(weeks)** | -45 | 0 |  | 1 | 28  (4) | 56  (8) | 84  (12) | 112  (16) | 140  (20) | 168  (24) | 182  (26) |  |
| **Allowance (± days)** | - | - | - | - | ±7 | ±7 | ±7 | ±7 | ±7 | ±7 | ±7 |  |
| **Outpatient/Inpatient** | Outpatient | In | In | In | Out | Out | Out | Out | Out | Out | Out | Out |
| **Consent acquisition** | ○ |  |  |  |  |  |  |  |  |  |  |  |
| **Subject background^b^** | ○ |  |  |  |  |  |  |  |  |  |  |  |
| **Urine pregnancy test** | ○ |  |  |  |  |  |  |  |  |  |  | ○ |
| **Infectious disease**  **or antibody tests** | ○ |  |  |  |  |  |  |  |  |  |  |  |
| **Medical examination** | ○ | ○ | ○ | ○ | ○ | ○ | ○ | ○ | ○ | ○ | ○ | ○ |
| **Vital signs^c^** | ○ | ○ | ○ | ○ | ○ | ○ | ○ | ○ | ○ | ○ | ○ | ○ |
| **EDSS/FS** | ○ |  |  |  | ○ | ○ | ○ | ○ | ○ | ○ |  | ○ |
| **Clinical tests (hematological test^d^, hematobiochemical test^e^, and urine test^f^)** | ○ | ○ |  | ○ | ○ | ○ | ○ | ○ | ○ | ○ |  | ○ |
| **12-lead ECG** | ○ | ○ |  |  |  | ○ |  | ○ |  | ○ |  | ○ |
| **Echocardiography** | ○ |  |  |  |  |  |  |  |  |  |  | ○ |
| **Chest or**  **abdominal X-ray test** | ○ |  |  |  |  |  |  |  |  |  |  | ○ |
| **Abdominal** **CT test** | ○ |  |  |  |  |  |  |  |  |  |  | ○ |
| **MRI^g^** | ○ |  |  |  |  |  | ○ |  |  | ○ |  | ○ |
| **Administration of investigational drug** |  |  | ○ |  | ○ | ○ | ○ | ○ | ○ |  |  |  |
| **Gene expression level measurement (RT-PCR)** |  | ○ |  | ○ |  |  |  |  |  | ○ |  | ○ |
| **Lymphocyte subsets, Th1, Th2, or Th17 cells^h^** |  | ○ |  | ○ |  |  |  |  |  | ○ |  | ○ |
| **Intestinal and oral microbiome analysis** | ○ |  |  |  |  |  |  |  |  | ○ |  | ○ |
| **C-SSRS** | ○ |  |  |  |  |  | ○ |  |  | ○ |  | ○ |
| **Adverse events**  **survey period** |  |  |  |  |  |  |  |  |  |  |  |  |
| **Concomitant drugs or**  **combination therapies** |  |  |  |  |  |  |  |  |  |  |  |  |

Abbreviations: C-SRSS, Columbia Suicide Severity Rating Scale; ECG, electrocardiogram; EDSS, Expanded Disability Status Scale; FS, functional disability scale; MRI, magnetic resonance imaging; RT-PCR, reverse transcription-polymerase chain reaction.

^a^Investigational drug can be assigned using a case enrollment system based on the results of the screening period.

^b^Including birth date, sex, body height, and body weight.

^c^Blood pressure, pulse rate, body temperature, and breathing rate; T1 vital signs were measured 2 ± 1 h after administration of the investigational drug and no time is specified for other vital sign measurements.

^d^Red blood cell count, platelet count, hemoglobin, hematocrit, white blood cell count, and differential white blood count (lymphocytes)

^e^Alanine aminotransferase, aspartate aminotransferase, gamma-glutamyl transpeptidase, total bilirubin, direct bilirubin, albumin, creatinine, amylase, total cholesterol, neutral fat, high density lipoprotein cholesterol, low density lipoprotein cholesterol, blood glucose, C-reactive protein, sodium, potassium, chloride, total protein, albumin-globulin ratio, blood urea nitrogen, creatine kinase and lactate dehydrogenase.

^f^Urine protein, urine glucose, urine white blood cells, and urine occult blood.

^g^MRI was performed at the National Center of Neurology and Psychiatry.

^h^This study was performed only on patients enrolled at the National Center of Neurology and Psychiatry.
